# Study on the mechanism of efficient extracellular expression of toxic streptomyces phospholipase D in *Brevibacillus choshinensis* under Mg^2+^ stress

**DOI:** 10.1186/s12934-022-01770-z

**Published:** 2022-03-19

**Authors:** Shaofeng Chen, Weide Xiong, Xurui Zhao, Weiyi Luo, Xuhui Yan, Yinghua Lu, Cuixue Chen, Xueping Ling

**Affiliations:** 1 Department of Chemical and Biochemical Engineering, College of Chemistry and Chemical Engineering, Xiamen University, Xiamen, 361005 People’s Republic of China; 2 The Key Laboratory for Chemical Biology of Fujian Province, (Xiamen University), Xiamen, People’s Republic of China; 3 Fujian Collaborative Innovation Center for Exploitation and Utilization of Marine Biological Resources, Xiamen, Fujian People’s Republic of China; 4 Department of Pharmacy, Xiamen Medical College, Xiamen, People’s Republic of China

**Keywords:** Phospholipase D, *Brevibacillus choshinensis*, Magnesium stress, P2 promoter, Ion perturbation

## Abstract

**Background:**

Phospholipase D (PLD) has significant advantages in the food and medicine industries due to its unique transphosphatidylation. However, the high heterologous expression of PLD is limited by its cytotoxicity. The present study sought to develop an efficient and extracellular expression system of PLD in the non-pathogenic *Brevibacillus choshinensis* (*B. choshinensis*).

**Results:**

The extracellular PLD was effectively expressed by the strong promoter (P2) under Mg^2+^ stress, with the highest activity of 10 U/mL. The inductively coupled plasma–mass spectrometry (ICP-MS) results elucidated that the over-expression of PLD by P2 promoter without Mg^2+^ stress induced the ionic homeostasis perturbation caused by the highly enhanced Ca^2+^ influx, leading to cell injury or death. Under Mg^2+^ stress, Ca^2+^ influx was significantly inhibited, and the strengths of P2 promoter and HWP gene expression were weakened. The study results revealed that the mechanism of Mg^2+^ induced cell growth protection and PLD expression might be related to the lowered strength of PLD expression by P2 promoter repression to meet with the secretion efficiency of *B. choshinensis*, and the redistribution of intracellular ions accompanied by decreased Ca^2+^ influx.

**Conclusions:**

The PLD production was highly improved under Mg^2+^ stress. By ICP-MS and qPCR analysis combined with other results, the mechanism of the efficient extracellular PLD expression under Mg^2+^ stress was demonstrated. The relatively low-speed PLD expression during cell growth alleviated cell growth inhibition and profoundly improved PLD production. These results provided a potential approach for the large-scale production of extracellular PLD and novel insights into PLD function.

**Supplementary Information:**

The online version contains supplementary material available at 10.1186/s12934-022-01770-z.

## Background

Phospholipase D (PLD, EC 3.1.4.4) could synthesize novel medicinal bioactive phospholipids [1] by introducing its functional polar heads into the common phospholipids through the transphosphatidyl reaction. The phosphatidyl derivatives, including phosphatidylglycerol (PG) [2, 3], phosphatidyl ethanolamine (PE) [4], phosphatidylserine (PS) [5–7], and phosphatidylinositol (PI) [8] have significant advantages in the food and medicine industries. For instance, PS, the primary component of nerves in the brain [9–11], could exert a positive effect on the age-related memory disorders [12], Alzheimer’s disease [13], and attention deficit and hyperactivity disorder (ADHD) [14]. In recent years, numerous novel phospholipids with anti-cancer and antioxidant activities have been developed and utilized by PLD, such as phosphatidyl-batilol [15], phosphatidyl glucose [16], phosphatidyl serinol [17], indicating the wide application prospect of PLD.

Although PLD is abundant in nature, the low content of wild-type PLD and difficulty in extraction have affected its industrial production to meet the market demand [10]. Therefore, many researchers have attempted to isolate strains with high PLD production [5] or develop the PLD heterologous expression systems. So far, PLDs from different sources have been successfully expressed in *Escherichia coli* (*E. coli*) [18–20], *Streptomyces lividans* [21, 22], *Pichia pastoris* [23], and *Bacillus. subtilis* [24], but their extracellular synthesis with high-efficiency is still strenuous due to their cytotoxicity [20, 21]. According to reports, the highest PLD production in *E. coli* Top10 was 1100 U/mL [25], but it was intracellular expression. The extracellular expression systems have recently gained increased attention due to the easy separation and production of extracellular PLD. However, the reported highest PLD level (under 10^2^ U/mL) could not meet the industrial requirements. Therefore, extensive research on the extracellular expression protocols is highly warranted.

The expression system of *B. choshinensis* (formerly *Bacillus brevis* HPD31) is more convenient for secreted active proteins production due to efficient extracellular protein secretion [26, 27]. This system has also been extensively used to produce various foreign proteins, including cellulose [28], metalloproteinase [29], and nanobody [30]. Furthermore, *B. choshinensis* has been engineered to produce nearly negligible extracellular proteases ensuring the integrity of the foreign protein produced and eliminating spore forming capacity. This strain has also been confirmed to be nonpathogenic, with significant potential in the food microbiology industry [26]. Therefore, in this study, the *B. choshinensis* expression system was employed for PLD production.

Accumulating studies have reported that a substantial heterologous expression of PLD might cause significant damage to the cell. Based on this report, a weak promoter P5 was first used to express PLD extracellularly by *B. choshinensis*. After the PLD gene was successfully expressed in *B. choshinensis*, the promoter was replaced with a stronger promoter P2 to improve the PLD production, which showed significant increase under magnesium ion containing medium. Moreover, the possible regulatory mechanisms from the level of gene transcription to intracellular ion distribution were explored. The results showed that *B. choshinensis* could be a potential approach for expressing substantial amounts of heterologous PLD.

## Results

### Expression of PLD by the recombinant *B. choshinensis* with two different promoters

In this study, two different recombinant *B. choshinensis* expressing phospholipase D were successfully constructed by using two different plasmid vectors, namely pNY326 (weak P5 promoter) and pNCMO2 (strong P2 promoter).

**Fig. 1:**
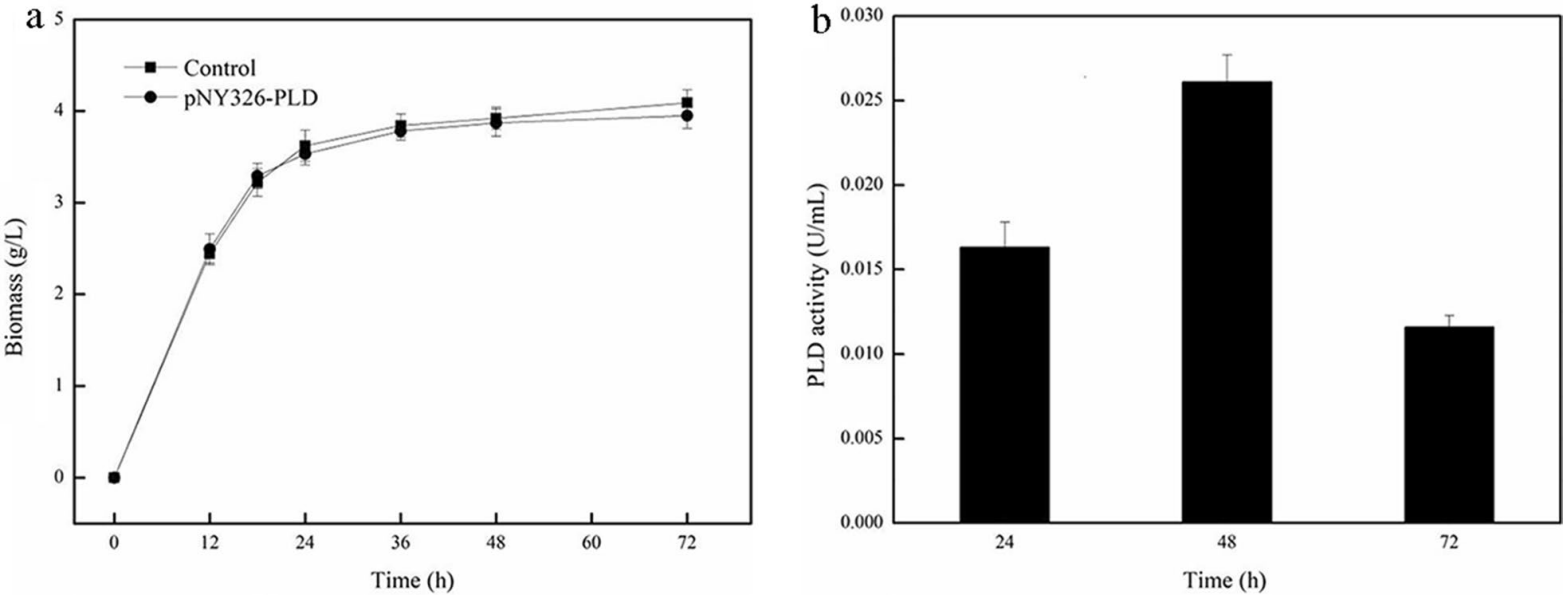
Growth curve and PLD activity of *B. choshinensis*/pNY326-PLD in TM medium. **a** Growth curve, the control group is the strain without plasmid. **b** PLD activity

When the transformed strains were cultured on plate, the culture showed the presence of *B. choshinensis/*pNY326-PLD, while not any trace of *B. choshinensis/*PNCMO2-PLD was detected. The promoters of the two expression vectors had significant differences in strength. As depicted in Fig. 1, *B. choshinensis/*pNY326-PLD containing P5 promoter was consistent with the growth of the control group (Fig. 1a) and expressed a small amount of PLD (Fig. 1b), which indicated that the low expression of PLD by *B. choshinensis* under weak constitutive promoter P5 would not damage the host cell. It was speculated that the strength of the P2 promoter in pNCMO2-PLD was much greater than P5 promoter [26] in pNY326-PLD, inducing strong expression of PLD, but high PLD expression severely inhibited cell growth due to its cytotoxicity. Therefore, for improving PLD expression, two problems need to be further considered: one is how to reduce the strength of the P2 promoter, and the other is how to reduce the adverse effects of PLD on cell growth.

**Fig. 2:**
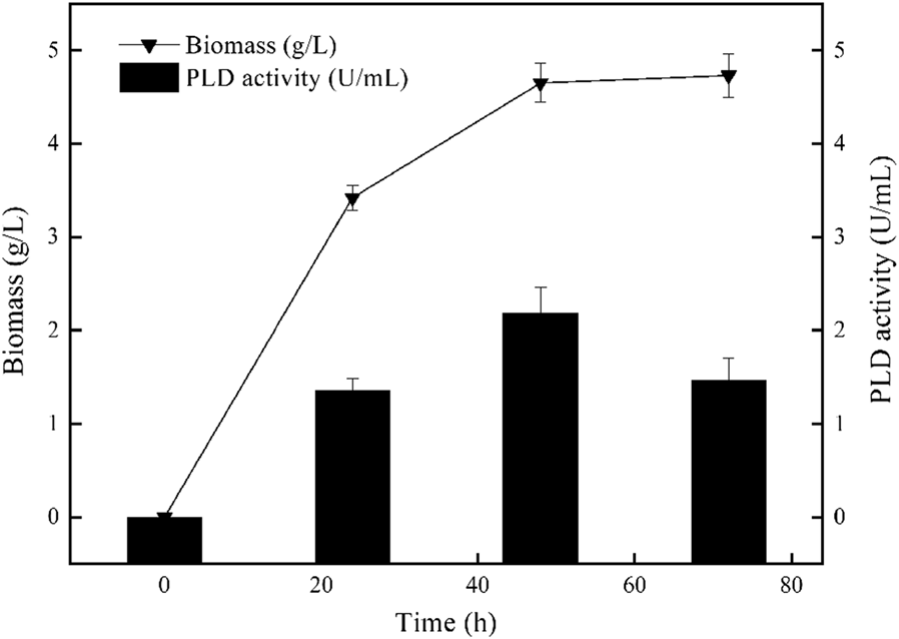
Growth curve and extracellular PLD expression of *B. choshinensis*/pNCMO2-PLD in TM medium with Mg^2+^

**Fig. 3:**
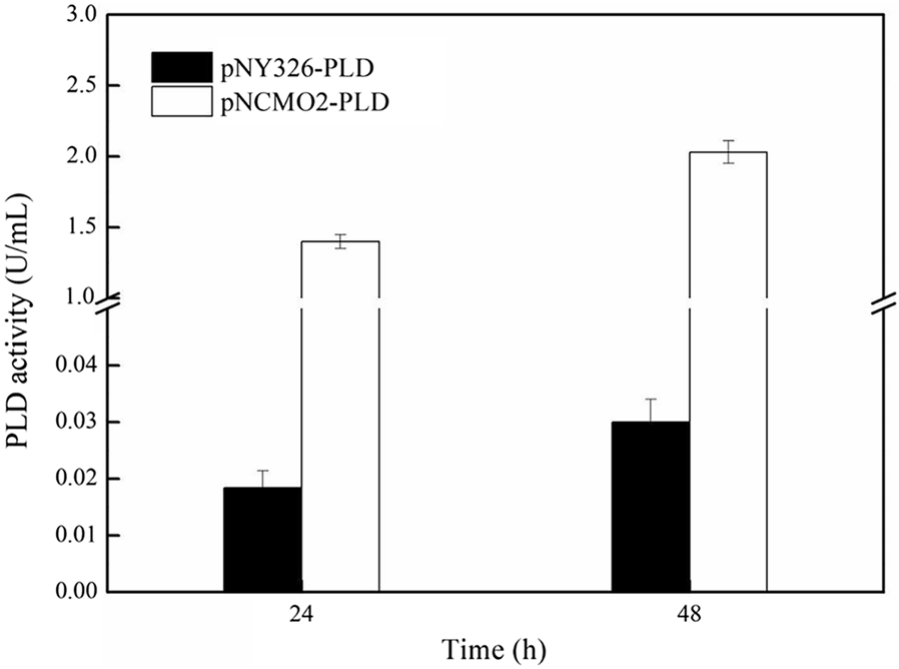
Effect of P5 and P2 promoter on the expression of PLD. *B. choshinensis*/pNY326-PLD (filled bar), pNCMO2-PLD (open bar)

According to reports, magnesium ions could specifically inhibit the activity of P2 promoter [31] and alleviate the cytotoxicity of PLD [20]. Therefore, in this study, 60 mM of MgSO_4_ was added to the transformation plate to culture *B. choshinensis/*pNCMO2-PLD. As depicted in Fig. 2, *B. choshinensis/*pNCMO2-PLD showed normal growth, with a significant increase in PLD production. Comparing the growth of the two recombinant cells (*B.choshinensis/*pNY326-PLD was cultured in the TM medium and *B.choshinensis*/pNCMO2-PLD was cultured in the TM medium with 60 mM MgSO_4_) (Fig. 3), the higher extracellular activity of PLD (2.03 U/mL) was obtained by the pNCMO2-PLD expression system, which was 67-fold higher than pNY326-PLD (0.03 U/mL). These results indicated that the strong promoter could enhance PLD expression under magnesium ion stress, which plays a vital role in PLD expression by *B.choshinensis*/pNCMO2-PLD. However, its mechanism needs further investigation.

### Determination of the culture medium

In the initial experiments, the TM medium was used to culture the cells. However, the recombinant cells showed unstable growth, suggesting that the TM medium was not optimal for PLD expression. Therefore, we first compared media, including 2SY, TM medium, and a fermentation medium from *B. choshinensis* to efficiently express the pullulanase [32] under 60 mM MgSO_4_, then chose the fermentation medium for further optimization. The results are depicted in Additional file 1: Fig. S1 and S2. After optimization, the modified fermentation medium contained 30.0 g/L glucose, 30.0 g/L beef extract, 25.0 g/L yeast extract, and 60 mM MgSO_4_, which increased PLD production by 7 times.

### Effect of metal ions on phospholipase D expression by *B. choshinensis*

**Fig. 4:**
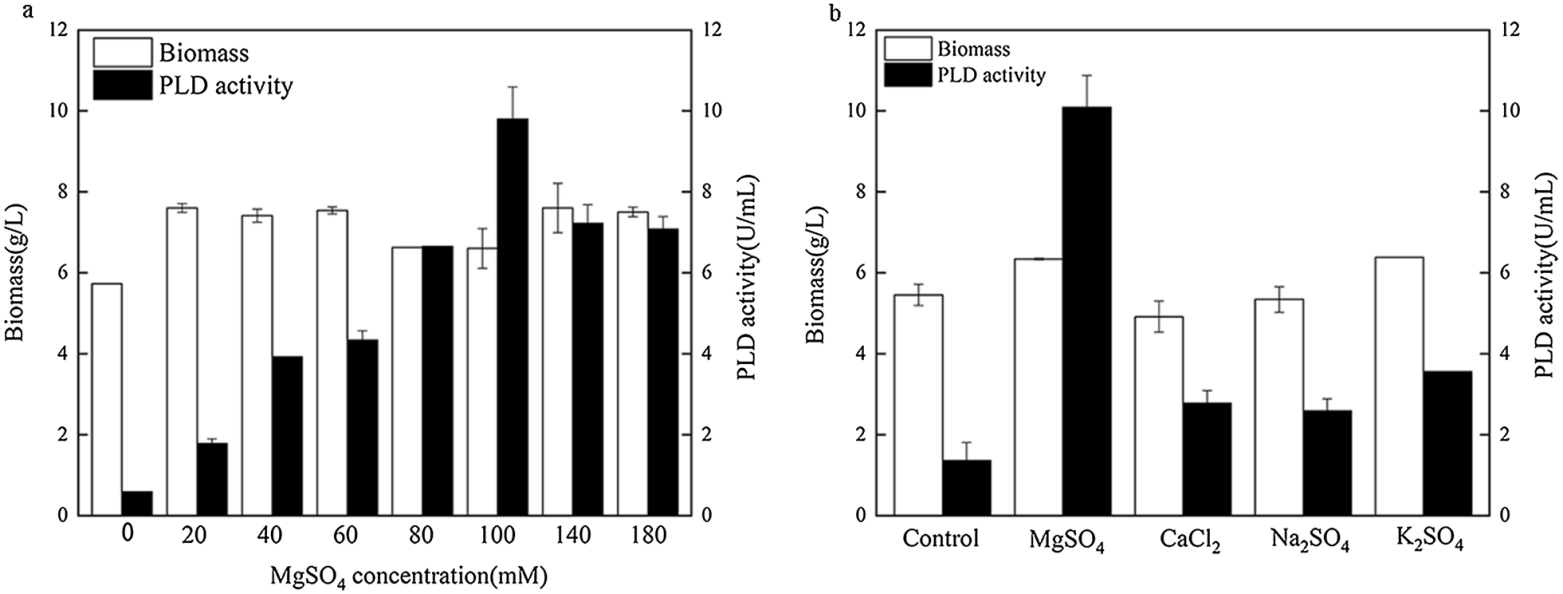
Effects of different conditions on the expression of PLD by *B. choshinensis*. **a** Different MgSO_4_ concentrations. **b** Different salts. Biomass (open bar), phospholipase D activity (filled bar)

The optimal concentration of Mg^2+^ was investigated because Mg^2+^ could increase PLD production in *B. choshinensis*. As shown in Fig. 4a, the biomass exhibited a slight increase at different Mg^2+^ concentration, while the PLD activity continued to increase until it reached a peak value of 9.8 U/mL at 100 mM Mg^2+^.

The result showed that magnesium ion had a positive promoting effect on PLD expression in *B. choshinensis*/pNCMO2-PLD, speculating similar effects from other metal ions. Therefore, the effects of Na^+^, K^+^, Ca^2+^, and Mg^2+^ on cell growth and PLD production were investigated in the modified fermentation medium. As depicted in Fig. 4b, only Mg^2+^ and K^+^ stimulated cell growth to some extent, while Ca^2+^ and Na^+^ inhibited cell growth. For PLD activity, all ions had promoting effect, but the growth was highly stimulated in the presence of Mg^2+^, reaching the highest activity of 10.09 U/mL. So, the modified fermentation medium with 100 mM MgSO_4_ (named as medium A) was used as the optimal medium in the following experiments. Also, the anions of Na_2_SO_4_, K_2_SO_4_, and MgSO_4_ groups were all SO_4_^2−^, but the PLD activity was significantly different, which indicated that anion was not the key factor for promoting the PLD activity.

### Analysis of secreted PLD production by SDS-PAGE

**Fig. 5:**
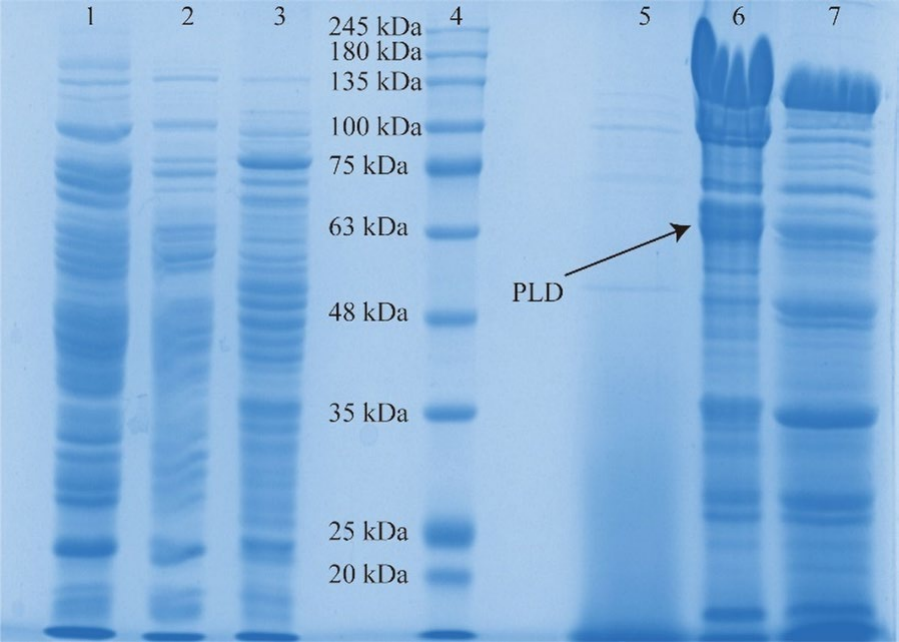
SDS-PAGE analysis of PLD expression. It was harvested at 48 h. Lane 4: protein marker; lanes 1,7: intracellular and extracellular protein of *B. choshinensis*/pNCMO2-PLD cultured in medium A without Mg^2+^; lanes 2,6: intracellular and extracellular protein of *B. choshinensis*/pNCMO2-PLD cultured in medium A; lanes 3,5: intracellular and extracellular protein of *B. choshinensis* cultured in medium A

In order to understand how Mg^2+^ affects PLD production, SDS-PAGE analysis was performed. As shown in Fig. 5, a band about 64 kDa in protein samples of *B. choshinensis/*pNCMO2-PLD was detected (lanes 1, 2, 6, 7), while there was no obvious band at 64 kDa in protein samples of *B. choshinensis* (lanes 3, 5). It indicated that PLD was successfully expressed in recombinant cells. Comparing extracellular protein samples showed in lane 6 and 7, Mg^2+^ induced more PLD expressed and secreted, which is in good agreement with the PLD activity value (Fig. 4a). Besides, expressing PLD could cause sever protein leakage (lanes 5, 6, 7) due to its cytotoxicity. Comparing intracellular protein samples showed in lane 1 and 2, there were PLD detected. It suggested PLD might accumulate in cells. Furthermore, lane 2 presented less PLD than lane 1, indicating more accumulated intracellular PLD could inhibit its extracellular expression and secretion, and Mg^2^ might hinder intracellular PLD to accumulate and benefit PLD expression and secretion.

### Ion perturbation in the PLD-imposed cells

Xiong et al. found that the PLD-imposed cells in *E. coli* underwent ion perturbations, resulting in cell injury or cell death. Na^+^ stress protects the PLD-imposed cells by improving oxidative phosphorylation through a positive change in the membrane potential via redistribution of Na^+^/K^+^ inside and outside the cell [25]. Therefore, *B. choshinensis/*pNCMO2 was cultured in medium A without Mg^2+^ (NP group), and *B. choshinensis*/pNCMO2-PLD was cultured in medium A (EPMg group) and without Mg^2+^ (EP group) to determine whether Mg^2+^ has a similar regulatory mechanism for PLD expression. The presence of different ions in cells (Na^+^, K^+^, Mg^2+^, Ca^2+^, Fe^2+/3+^, Zn^2+^ and Mn^2+^) were evaluated in the NP, EP and EPMg groups.

**Fig. 6:**
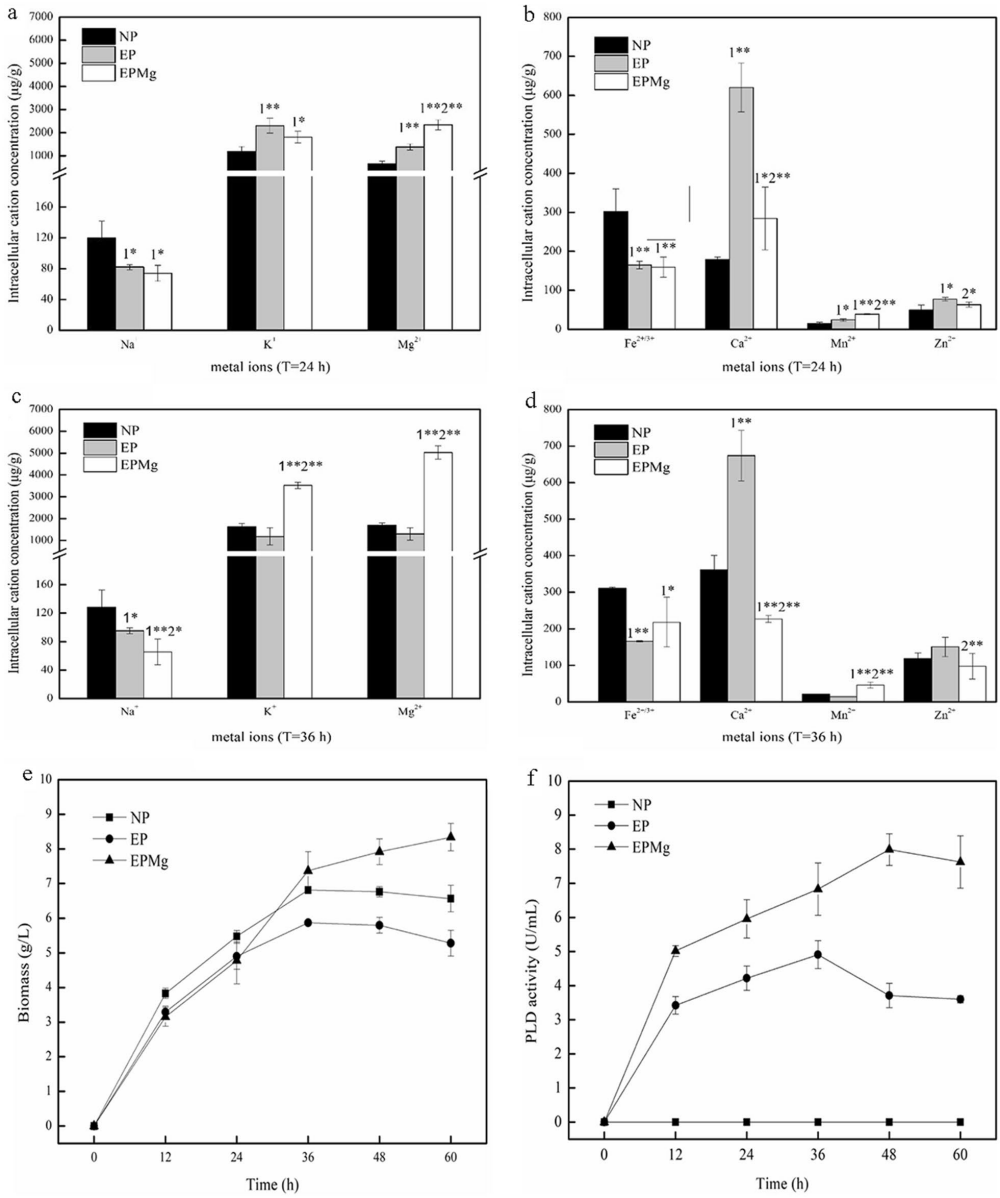
The concentration of ions. Intracellular Na^+^, K^+^, and Mg^2+^ (**a**), Ca^2+^, Fe^2+/3+^, Zn^2+^, and Mn^2+^ (**b**) concentrations in groups NP, EP, EPMg at 24 h. Intracellular Na^+^, K^+^, and Mg^2+^ (**c**), Ca^2+^, Fe^2+/3+^, Zn^2+^, and Mn^2+^ (**d**) concentrations in groups NP, EP, EPMg at 36 h. Time-course of biomass (**e**) and PLD activity (**f**) in groups NP, NPMg, EP, EPMg

We initially focused on ion distribution at 24 h (Fig. 6a and b) and found the same change trend in the three groups. For instance, [Na^+^] and [Fe^2+/3+^] decreased, while [K^+^], [Mg^2+^], [Ca^2+^], [Mn^2+^], and [Zn^2+^] increased, resulting in similar cell growth. Compared with the EP group, higher [Mg^2+^] and [Mn^2+^] with an increasing rate of 67.86% and 61.62%, and lower [K^+^] and [Ca^2+^] with a decreasing rate of 23.91% and 54.17% were observed in the EPMg group. At 36 h, most of the ions maintained a steady-state, while [Ca^2+^] and [Zn^2+^] showed an increase of 101.93% and 140.32% (Fig. 6c and d). Meanwhile, the expressed PLD exhibited significant changes in [K^+^], [Mg^2+^], and [Ca^2+^], suggesting that PLD expression might affect the cell growth status by influencing ion distribution. Under Mg^2+^ stress, [K^+^] and [Mg^2+^] increased by 198.55% and 287.83%, while [Na^+^] and [Ca^2+^] decreased by 31.08% and 66.31%, respectively. These results demonstrate that Mg^2+^ stress changes the regulation of some intracellular ions in the PLD-imposed cells, which might change the cell physiology status.

The changes of ion contents in the cells were not consistent at different growth periods under Mg^2+^ stress. Notably, [Ca^2+^] exhibited extraordinary change at 24 h and 36 h. Compared to the NP group, [Ca^2+^] of the EP group significantly increased. When Mg^2+^ stress was introduced to the medium (EPMg group), [Ca^2+^] reduced sharply, indicating that [Ca^2+^] might regulate cell growth. Comparing the changes of ions in three groups at 36 h, [K^+^] and [Mg^2+^] were found to have the lowest concentration in the EP group and the highest concentration in the EPMg group. On the contrary, [Ca^2+^] had the highest value in the EP group and the lowest value in the EPMg group. The combined results of Fig. 6e and f suggest that the addition of Mg^2+^ in the PLD-imposed cells could keep the cells with low [Ca^2+^], high [K^+^], and [Mg^2+^] contents, which is beneficial for cell growth.

The toxic effect of PLD on cells was minute due to the low level of PLD expression in the early growth stage. Therefore, the cell growth of both EP and EPMg groups was slightly lower than the NP group before 24 h (Fig. 6e). From 24 h, PLD expression started to increase to a great extent, which significantly changed the ion distribution of the experimental group, inducing cells showed significant different growth states. Of the three groups, the biomass in the EP group was the lowest at 36 h, which began to decline after 36 h. The biomass in the EPMg group surpassed the NP group at 36 h and continued to rise. As depicted in Fig. 6b and e, [Ca^2+^] of the EP group with the lowest growth state was significantly higher than the other two groups at 24 h and 36 h. However, the EPMg group with the best growth state at 36 h was significantly lower than the EP and NP groups, indicating that PLD significantly increased the intracellular [Ca^2+^] content, which was harmful to the cells. In contrast, the addition of Mg^2+^ could significantly inhibit the increase of Ca^2+^ induced by PLD expression to alleviate the toxic effect of PLD on cells, resulting in the continuous increase of cell growth and PLD expression. Meanwhile, the growth of *B. choshinensis* was inhibited under Ca^2+^ stress (Fig. 6a), indicating Ca^2+^ to be an essential factor affecting the growth of *B. choshinensis*.

**Fig. 7:**
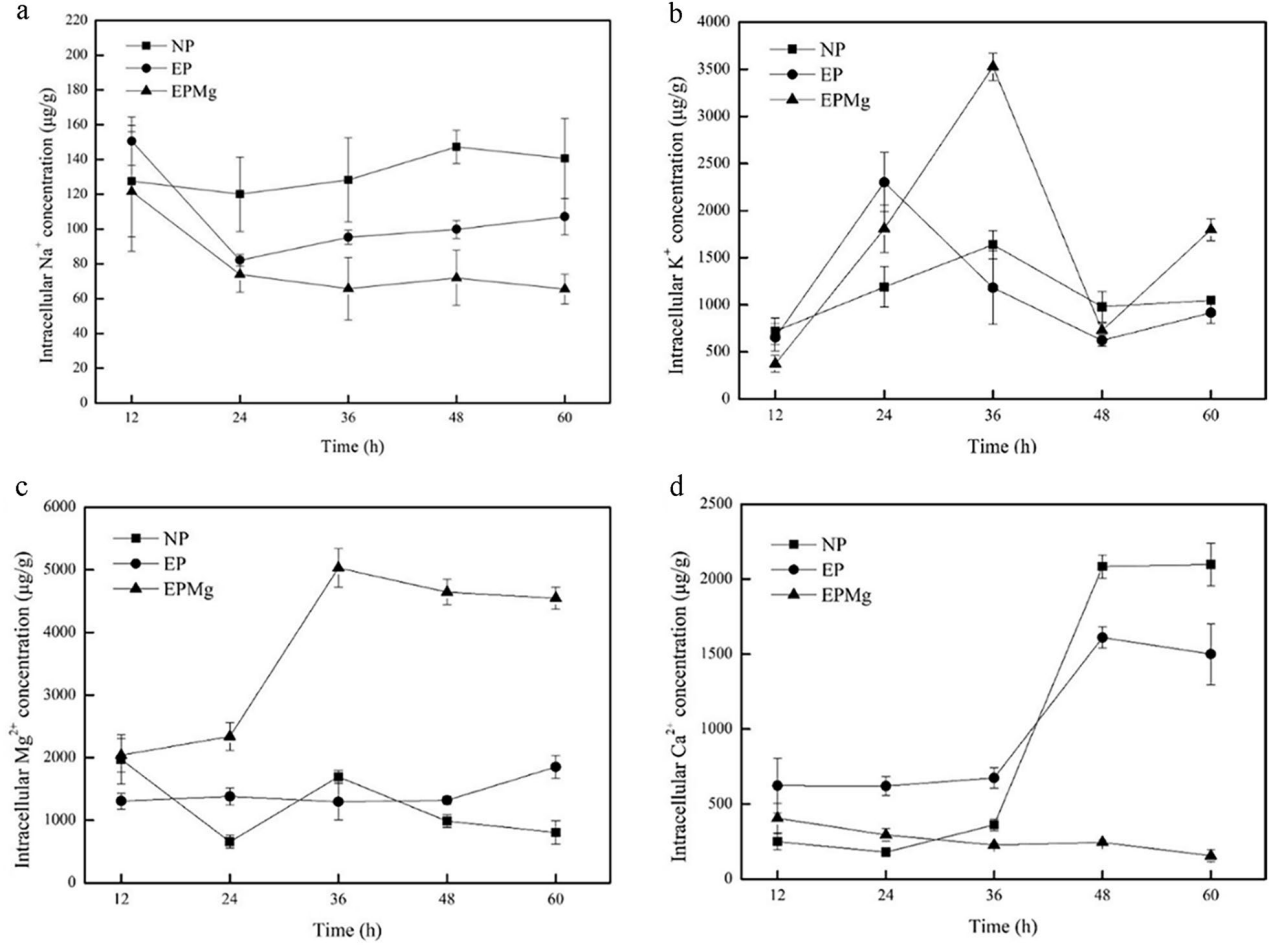
Curves of four ions under different conditions. **a** Intracellular [Na^+^]. **b** Intracellular [K^+^]. **c** Intracellular [Mg^2+^]. **d** Intracellular [Ca^2+^]

The intracellular concentrations of four ions (Na^+^, K^+^, Mg^2+^, Ca^2+^) were investigated from 12 to 60 h to further analyze the effect of ion changes. Compared with the NP group, [Na^+^] showed a similar decreasing trend at the first 24 h, while [K^+^] had a similar increase in the EP and EPMg groups (Fig. 7a and b). However, after 24 h, [K^+^] began to decrease accompanied by an increase in [Na^+^] content in the EP group, while in the EPMg group, [K^+^] continued to increase accompanied by a steady low [Na^+^]. In 24 h, the continued PLD expression in the cell caused K^+^ efflux and Na^+^ influx in the EP group, which was harmful to the cells [25]. However, the addition of Mg^2+^ could regulate the change in ions. As depicted in Fig. 7c, [Mg^2+^] in the EPMg group showed a higher growth rate than the other two groups. It is reported that Mg^2+^ could enhance the activity of Na^+^-K^+^-ATPase [33], thereby triggering K^+^ influx and Na^+^ efflux in the EPMg group, keeping [Na^+^] lower and [K^+^] higher inside the cells to promote cell growth. As depicted in Fig. 7d, [Ca^2+^] in the EP group with the lowest biomass had a high concentration. In contrast, the EPMg group with the highest biomass had low [Ca^2+^] during the whole process. Although low [Ca^2+^] was observed in the NP group before 24 h, it began to increase after 36 h, showing a sharp increase. [Ca^2+^] of the EP group simultaneously exhibited similar increasing trend after 36 h. As depicted in Fig. 6e, the EPMg group showed increased biomass, while the other two groups showed a decreased biomass after 36 h. The results suggested that the growth of *B. choshinensis* was coupled with the intracellular [Ca^2+^] and low intracellular [Ca^2+^] was beneficial for cell growth.

Xiong et al. expressed PLD in *E. coli* and found that it could cause a continuous influx of Na^+^, the outflow of K^+^, and increase of intracellular Ca^2+^, while cations stress (especially Na^+^) alleviated cell growth inhibition and profoundly increased PLD production by redistributing the ions inside and outside the cell. The effect of salt stress on PLD expression was consistent with our study results. However, PLD expression in *B. choshinensis* showed a different phenomenon without any continuous influx of Na^+^. Therefore, it was speculated that the effect of Mg^2+^-induced protection on cell growth and PLD expression might be related to the P2 promoter expression.

### Transcription level of PLD and HWP genes in *B. choshinensis*/pNCMO2-PLD under Mg^2+^ stress

HPD31 Wall Protein (HWP) is the cell wall protein secreted by *B. choshinensis,* whose expression could also be regulated by the P2 promoter [34, 35]. Therefore, the analysis of HWP expression level could further reflect the level of P2 promoter strength.

**Fig. 8:**
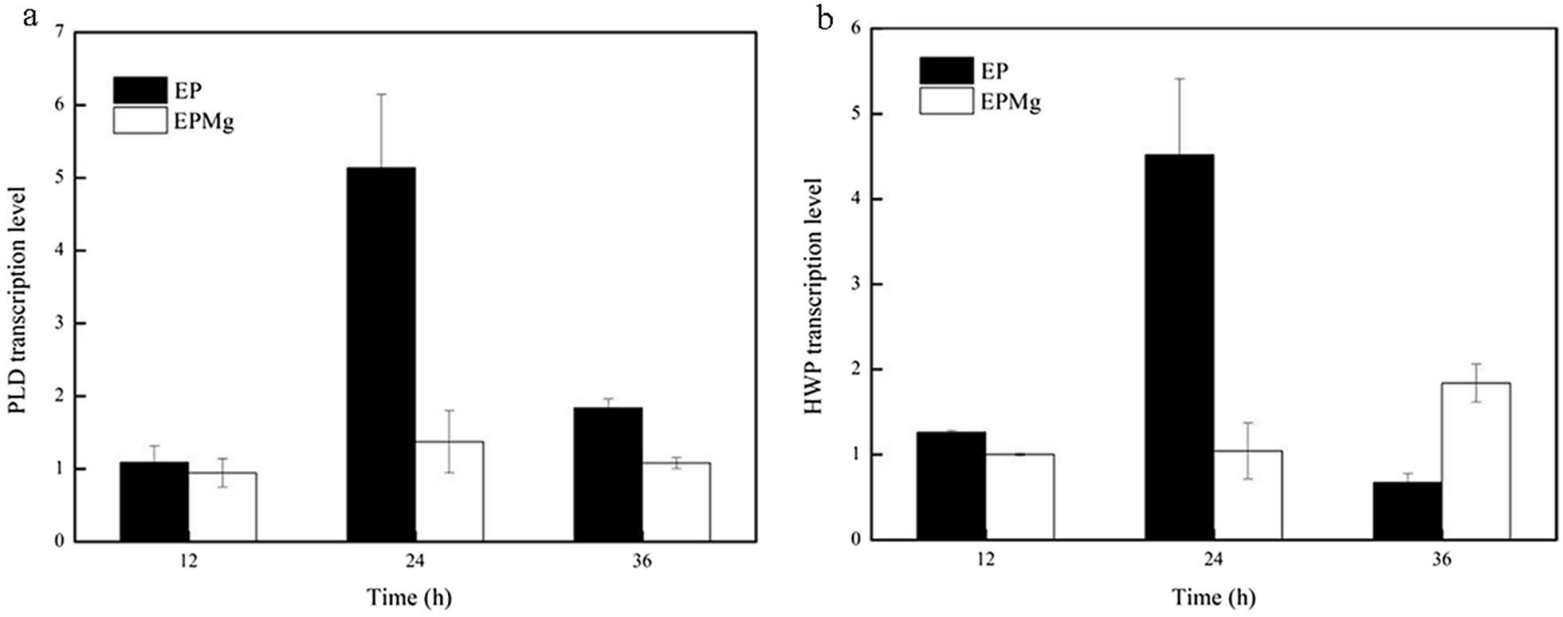
Transcription levels of phospholipase d and HWP genes in *B. choshinensis/*pNCMO2-PLD. **a** Phospholipase D. **b** HWP

As depicted in Fig. 8a and b, the transcriptional levels of PLD and HWP genes in the EP group were consistent with the EPMg group at 12 h, but both increased sharply in the EP group at 24 h, which were 5.14 times and 4.52 times higher than at 12 h, respectively. Subsequently, the transcription level of PLD and HWP decreased significantly at 36 h. It might be attributed to PLD accumulation which induced a toxic effect on cell growth (Fig. 6e), resulting in the death of the recombinant strain and decreased gene expression. As for the EPMg group, the transcription levels of PLD and HWP exhibited minute changes at 24 h and 36 h compared to 12 h. The results indicated that Mg^2+^ reduced the strength of the P2 promoter reflected by the decreased transcriptional level of the HWP gene, further maintaining the PLD gene at a relatively low transcription level during cell growth. Therefore, the toxic effect caused by highly expressed PLD was inhibited, which was conducive to the continuous cell growth and expression of PLD in *B. choshinensis* (Fig. 7a and b)*.*

As depicted in Fig. 6e, cell growth in three groups was consistent before 24 h, but it changed after 24 h. The biomass of the NP and EP groups gradually declined after 36 h, showing the lowest growth state in the EP group. In contrast, the biomass of the EPMg group significantly increased from 24 to 36 h, and continued to increase after 36 h until it reached a higher biomass state than the NP and EP groups after 36 h. The result demonstrated that the decreased growth of *B. choshinensis* by PLD expression was due to its toxicity, while the addition of Mg^2+^ attenuated the toxic effect of PLD on cells, exhibiting a positive effect on the growth of *B. choshinensis*. As depicted in Fig. 6f, the PLD activity of the EPMg group was higher than the EP group during the entire growth process, indicating that Mg^2+^ effectively promoted the expression of PLD. Besides, PLD activity in the EP group decreased after 36 h, showing the same change in its biomass. Furthermore, the analyzed cells began to die after 36 h due to the high toxicity of PLD expression, which might cause the intracellular protease leakage leading to the degradation of extracellular PLD.

## Discussion

For extracellular expression, Huang et al. applied a series method including screening signal peptides, selecting plasmids, and optimizing the sequences of the ribosome-binding site and the spacer region to get the PLD production of 24.2 U/mL in flask which was higher than other reported extracellular PLD production in recent years [36]. But it didn’t investigate the mechanism of extracellular PLD expression. For intracellular expression, Xiong et al. reported the highest PLD production (1100 U/mL) in 5-L fermenter [25]. Although it was much higher than the reported extracellular PLD production, the intracellular production is inconvenient for PLD separation. The present study first successfully constructed the extracelluar expression system which reached a higher PLD activity of 10.09 U/mL, and further analyzed the mechanism of extracellular PLD expression under the stress of high concentration Mg^2+^.

Studies have elucidated the mechanism underlying the toxicity of PLD overexpression to cells and protection of the PLD-imposed cells by Mg^2+^. The abnormal cellular physiology was mediated by the cytotoxicity of PLD, especially due to the imbalance of intracellular ionic homeostasis. Of the above cases, Ca^2+^ significantly affected cellular physiology. Ca^2+^ is a highly versatile signaling molecule controlling numerous functions during the entire life cycle of cells [37, 38] and a powerful uncoupling agent, with a high affinity to the electron transfer system. Mitochondrial calcium overloaded the decoupled oxidative phosphorylation and hindered energy production [39], resulting in reduced and disordered cell functions [40, 41]. Similarly, Tomasz Wegierski has reported that TRPP2, the ion channel mutated in the autosomal dominant polycystic kidney disease (ADPKD), could protect cells from apoptosis by lowering Ca^2+^ concentration in the endoplasmic reticulum (ER) [42]. These studies suggested that excessive intracellular [Ca^2+^] could cause damage to the cells, even cell death. Besides, [Mg^2+^] needs further attention as the significant decrease of [Ca^2+^] was accompanied by the significant increase of [Mg^2+^] in the EPMg group, which was conducive to cell recovery and growth. The increase of [Ca^2+^] by PLD expression in *B. choshinensis* was effectively alleviated under Mg^2+^ stress, which redistributed the intracellular ions, especially decreased [Ca^2+^] influx, and increased the growth status of *B. choshinensis*.

The secretion promoted by the signal peptide in the vector pNCMO2-PLD, derived from the MWP of *Bacillus brevis* 47 [26], led the preprotein of PLD through the cytoplasmic membrane and then folded as mature proteins [35]. Gong reported that the expression of *Stv. Cinnamoneus* PLD in *B. subtilis* resulted in the production of inclusion bodies due to excessive promoter strength [43]. Huang et al. reported that during the expression of *Streptomyces racemochromogenes* PLD in *B. subtilis* WB600, the extracellular PLD activity controlled by the highest-strength P43 promoter was lower than the relatively weak PHpall promoter [44]. Moreover, the signal peptides with different secretion folding efficiency caused different extracellular PLD activity. These results suggested that the matching degree plays a pivotal role between the promoter strength and secretion efficiency of the signal peptide. When pullulanase was expressed by *B. choshinensis*, it was found that Mg^2+^ reduced the transcription level of the pullulanase gene, leading to the extracellular secretion of pullulanase in the correct form after folding [45]. The effect of Mg^2+^ on PLD in this study was consistent with its effects on pullulanase expression, indicating similar mechanism. In EP group, the P2 promoter exhibited a high transcriptional level of the PLD gene, leading to a higher protein synthesis and hasty cytotoxicity. As for the EPMg group, Mg^2+^ specifically inhibited the P2 promoter activity to maintain an appropriate transcriptional level during the growth stage, which ensured that PLD was properly secreted into the culture medium, thereby the cytotoxicity induced by high PLD expression was alleviated.

Proteins may misfold resulting accumulation in cells when the secretion could not match the expression [46]. Without Mg^2+^ stress, more PLD accumulated in cells (Fig. 5) and acquired lower PLD activity (Fig. 3) which provided evidence for the existence of misfolded PLD to a certain extent. Over-expression of PLD under the control of P2 promoter overloaded the protein secretion pathway, inducing incorrect forms of PLD and reducing its expression. It is speculated that high extracellular PLD expression has a connection with protein secretion efficiency and its correct folding. Mg^2+^ inhibited PLD expression in early stage to promote secretion efficiency matching expression and reduce PLD accumulation in cells. Therefore, cells could keep a continuous growth to produce PLD. However, there was a large amount of protein leakage accompanied by the PLD expression and secretion. It could also be meaningful for further research on PLD by exploring how to reduce the impact of PLD on cell leakage.

## Conclusions

To the best of our knowledge, this is the first study to report PLD expression in *B. choshinensis*. Studies showed a potential extracellular expression system with high PLD expression for industrial production. However, comparing to other host, low biomass limits the production, which suggests it requires the cultivation in bioreactors for higher production. And the mechanism behind PLD expression and secretion wouldn’t be fully elucidated. Overall, further investigation is necessitated to identify the underlying mechanism behind Mg^2+^-induced protection of the PLD-imposed cells by omics. This could be an alternative approach to meet the needs for economically viable production of rare bioactive phospholipids.

## Materials and methods

### Materials and strains

*Brevibacillus choshinensis* (Takara Bio Inc.) was used to express phospholipase D. Plasmid pNY326 and pNCMO2 (Takara Bio Inc.) were used as the expression vector. The strain *E. coli* JM109 (Takara Bio Inc.) was used as the cloning host. Other chemicals, unless otherwise noted, were purchased from Sinopharm Chemical Reagent Co. Ltd. (SCRC; Shanghai, China).

### Cultivation medium and conditions

*E. coli* JM109 was cultured on the Luria broth (LB) medium containing 10.0 g/L tryptone, 5.0 g/L yeast extract, and 10.0 g/L NaCl, with the introduction of 50 mg/L Ampicillin (Amp) to each culture. In the shake-flask culture, the primary inoculum of *B. choshinensis* was grown for 24 h in the 250 mL baffled flasks containing 50 mL of TM medium at 30 ℃ and 120 rpm in a shaker until OD 600 = 2 ~ 3. Subsequently, the seed culture (0.5 mL) was diluted to 50 mL of medium A in a 250 mL shake flask at 30 ℃ and 120 rpm. The TM medium contained 10.0 g/L glucose, 10.0 g/L tryptone, 5.0 g/L beef extract, 2.0 g/L yeast extract, 10.0 mg/L FeSO_4_·7H_2_O, 10.0 mg/L MnSO_4_·4H_2_O, and 1.0 mg/L ZnSO_4_ 7H_2_O. Medium A contained: 30 g/L glucose, 30.0 g/L beef extract, 25.0 g/L yeast extract, and 100 mM MgSO_4_ (It was the optimum concentration after optimization). The contents were modified according to the medium reported by Chun Zou et al. [32].

### Construction of two phospholipase D expression vectors

The PLD gene from *Streptomyces antibioticus* (Genebank NO. D16444, 64 kDa) with removed signal peptide was synthesized by Sangon Biotech (pUC57-PLD anti). The PLD gene was amplified by PCR using the primers 5ʹ-TGCTCTAGAGCGGACACACCGCCCACC-3ʹ, containing an Xba I site, 5ʹ-CCGGAATTCTCAGCCCGCCTGGCGAGCCGGGC-3ʹ, containing an *Eco*R I site, and the plasmid pUC57-PLD anti as the template. The product was ligated into the vector pNY326 (P5 promoter) via T4 ligase (Takara Bio Inc.), then the plasmid pNY326-PLD was transformed into *B. choshinensis*. As for another expression vector, the PLD gene fragment amplified by PCR was ligated into the vector pNCMO2 (P2 promoter) via T4 ligase. The hybridized fragment was transformed into *E. coli* JM109. After being confirmed by DNA sequencing, the plasmid obtained from the transformant was transformed into *B. choshinensis* through New Tris-PEG method (Takara Bio, DaLian, China). Finally, these two recombinant strains (*B.choshinensis*/pNCMO2-PLD and *B.choshinensis*/pNY326-PLD) were used for phospholipase D expression.

### Determination of biomass

Cell growth was monitored by measuring the optical density of the culture broth at 600 nm using a microplate reader. Accurately, 4 mL of the culture broth was pelleted by centrifugation at 13,000×*g* for 10 min to determine the dry cell weight (DCW). The pellet was resuspended in 0.9% (w/v) NaCl and re-pelleted by centrifugation. Later, it was frozen by liquid nitrogen for 1 min, which was immediately freeze-dried to constant weight by a vacuum freeze drier. Finally, the biomass standard curve was plotted according to OD_600_ and DCW values.

### Enzyme assay

The PLD activity was determined using phosphatidylcholine as a substrate according to the previously described method [20]. One unit of phospholipase D activity was defined as the amount of enzyme required to release 1 μmol choline per minute from phosphatidylcholine under the specified conditions.

### SDS-PAGE analysis

The celles harvested at 48 h were separated into two parts by centrifugation: the medium supernatant and the cell pellets. After pretreated, the extracellular and intracellular protein samples were added with 4 × SDS-PAGE loading buffer with DTT (Solarbio life science, Beijing, China) and boiled for 10 min, respectively. The proteins were separated in 10% SDS-PAGE. ColorMixed Protein Marker 5-245KD (Solarbio life science, Beijing, China) was used to determine the apparent molecular weight of separated proteins. Proteins were visualized with Coomassie Brilliant Blue.

### ICP-MS

*B. choshinensis* was cultured in 500 mL shake flasks. 20 mL of the culture broth was pelleted by centrifugation at 6000×*g* for 20 min at different time intervals. The pellet was resuspended in 1 × PBS, and re-pelleted by centrifugation for three times, then dried to a constant weight. The dried cells with 8 mL of nitric acid were completely digested by microwave digestion apparatus CEM MARS6 (CEM, America). Later, the liquid was completely evaporated and the leftover was re-dissolved in 2% nitric acid. The liquid sample was filtered, and then investigated under an inductively coupled plasma mass spectrometry (ICP-MS) (Agilent Technologies, America).

### Real-time quantitative PCR

Total RNA was extracted using the TaKaRa MiniBEST Universal RNA Extraction Kit (Takara Bio, DaLian, China), following the manufacturer’s instructions. The extracted RNA was used as the template for complementary DNA (cDNA) synthesis. The first-strand cDNA was synthesized using *EasyScript*^®^. All-in-One First-Strand cDNA Synthesis SuperMix for qPCR (One-Step gDNA Removal) (TransGen Biotech, BeiJing, China), following the manufacturer’s instructions.

The expression levels of the two genes encoding PLD and HWP were evaluated. The 16S rRNA gene was used as an internal control to normalize the results. The sequences of the primers used for quantitative real-time PCR (qPCR) are listed in Table 1. The qPCR analyses were performed using an ABI StepOne Real-Time PCR System (Applied Biosystems, SanMateo, CA, USA) coupled with TransStart Top Green qPCR SuperMix (+ DyeI/ + Dye II) (TransGen Biotech, Beijing, China). Amplification was performed in a 20 μL of mixture containing 10 μL of 2 × *TransStart*^®^ Top Green qPCR SuperMix (+ Dye I/ + Dye II), 0.4 μL of forward primer, 0.4 μL of reverse primer, 1 μL of cDNA, and 8.2 μL of nuclease-free water. The cycling program for qPCR was as follows: 94 °C for 30 s, followed by 40 cycles of 94 °C for 5 s, 60 °C for 30 s, then dissociation. The data were analyzed by using the 2^−ΔΔCT^ model [47].

**Table 1:** Primer sequences used in qPCR amplification

Gene	Primer	Sequence (5′–3′)
phospholipase D	phospholipase D-F	GGCGTGGGCATCAAGGAGT
	phospholipase D-R	CGGCGTTGGTGTTGTCGTG
HWP	HWP-F	CTCGCTTTGACTATGTACTGG
	HWP-R	TTTGGTGCCGTGACTACTTC
16S rRNA	16S rRNA-F	TCGTGTCGTGAGATGTTG
	16S rRNA-R	CCTTCCTCCGTCTTGTC

## Supplementary Information

**Additional file 1: Table S1.** Ingredients comparison of three mediums. **Fig. S1.** Effects of three mediums on B. choshinensis /pNCMO2-PLD cell growth (A) and PLD enzyme activity (B). All the medium contained 60 mM MgSO4. Fig. S2. Comparison of cell growth and phospholipase D production using A. different beef extract concentrations; B. different yeast extract concentrations; Biomass (open bar), PLD activity (filled bar). **Fig. S3.** Identification of reconstructed plasmid pNY326-PLD. Lane M: DNA marker; lanes pNY326-PLD: Sample of recombinant plasmid after Xba I and EcoR I double enzyme digestion. **Fig. S4.** Identification of reconstructed plasmid pNCMO2-PLD. Lane Marker: DNA marker; lanes 1-6: Sample of recombinant plasmid after Xba I and EcoR I double.** Fig. S5.** Transformation results of B. choshinensis under different conditions. Transformation of pNY326-PLD in TM with neomycin (A); transformation of pNCMO2-PLD in TM with neomycin (B); transformation of pNCMO2-PLD in TM with neomycin and Mg2+.enzyme digestion. **Fig. S6.** Identification of B. choshinensis/pNCMO2-PLD. Lane Marker: DNA marker; lanes 1-3: Sample of recombinant plasmid from the recombinant strains after Xba I and EcoR I double enzyme digestion.

## Data Availability

The data supporting the results of this article are included with the article.

## Abbreviations

PLD: Phospholipase D
*B. choshinensis*: *Brevibacillus choshinensis*
ICP-MS: Inductively coupled plasma–mass spectrometry
PG: Phosphatidylglycerol
PE: Phosphatidyl ethanolamine
PS: Phosphatidylserine
PI: Phosphatidylinositol
ADHD: Attention deficit and hyperactivity disorder
*E. coli*: *Escherichia coli*
HWP: HPD31 Wall Protein
ADPKD: Autosomal dominant polycystic kidney disease
ER: Endoplasmic reticulum
LB: Luria broth
Amp: Ampicillin
DCW: Dry cell weight
w/v: Weight/volume
cDNA: Complementary DNA
qPCR: Quantitative real-time
