# Supplementary material for: Study on the mechanism of efficient extracellular expression of toxic streptomyces phospholipase D in Brevibacillus choshinensis under Mg2+ stress
Source: Microb Cell Fact. 2022 Mar 19;21:41. doi: 10.1186/s12934-022-01770-z (PMC8933894; doi:10.1186/s12934-022-01770-z)
Supplement: Supplementary file 1 — Additional file 1: Table S1. Ingredients comparison of three mediums. Fig. S1. Effects of three mediums on B. choshinensis /pNCMO2-PLD cell growth (A) and PLD enzyme activity (B). All the medium contained 60 mM MgSO4. Fig. S2. Comparison of cell growth and phospholipase D production using A. different beef extract concentrations; B. different yeast extract concentrations; Biomass (open bar), PLD activity (filled bar). Fig. S3. Identification of reconstructed plasmid pNY326-PLD. Lane M: DNA marker; lanes pNY326-PLD: Sample of recombinant plasmid after Xba I and EcoR I double enzyme digestion. Fig. S4. Identification of reconstructed plasmid pNCMO2-PLD. Lane Marker: DNA marker; lanes 1-6: Sample of recombinant plasmid after Xba I and EcoR I double. Fig. S5. Transformation results of B. choshinensis under different conditions. Transformation of pNY326-PLD in TM with neomycin (A); transformation of pNCMO2-PLD in TM with neomycin (B); transformation of pNCMO2-PLD in TM with neomycin and Mg2+.enzyme digestion. Fig. S6. Identification of B. choshinensis/pNCMO2-PLD. Lane Marker: DNA marker; lanes 1-3: Sample of recombinant plasmid from the recombinant strains after Xba I and EcoR I double enzyme digestion. [file 12934_2022_1770_MOESM1_ESM.doc]

##### Additional file 1

**Table S1 Ingredients comparison of three mediums**

| Medium | Carbon source | | Nitrogen source | |
| --- | --- | --- | --- | --- |
|  | species | concentration（g/L） | species | concentration（g/L） |
| 2SY | glucose | 20 | tryptone yeast extract | 40  5 |
| TM | glucose | 10 | tryptone  beef extract yeast extract | 10  5  2 |
| Fermentation medium | glucose | 27.6 | beef extract yeast extract | 28  5 |


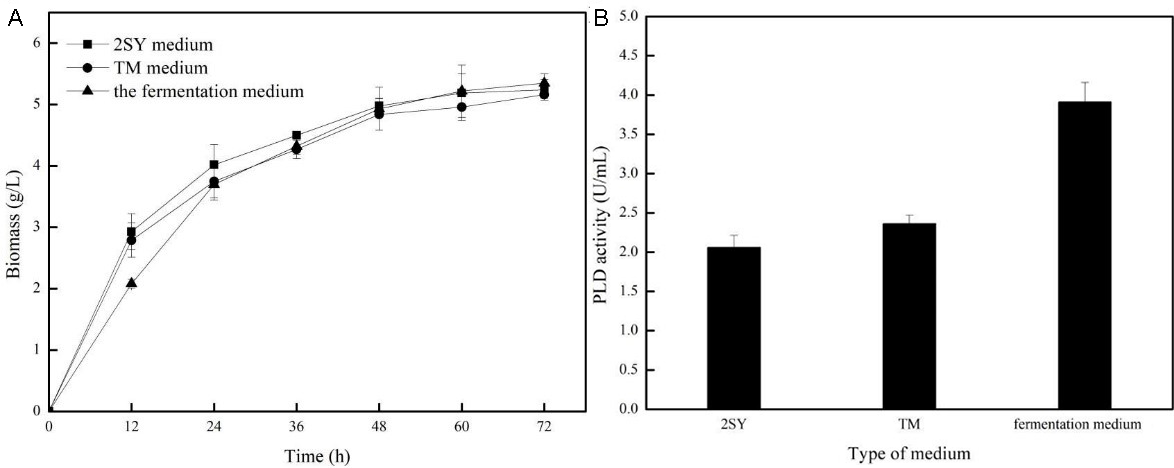


**Fig. S1** Effects of three mediums on *B. choshinensis* /pNCMO2-PLD cell growth (A) and PLD enzyme activity (B). All the medium contained 60 mM MgSO_4_.


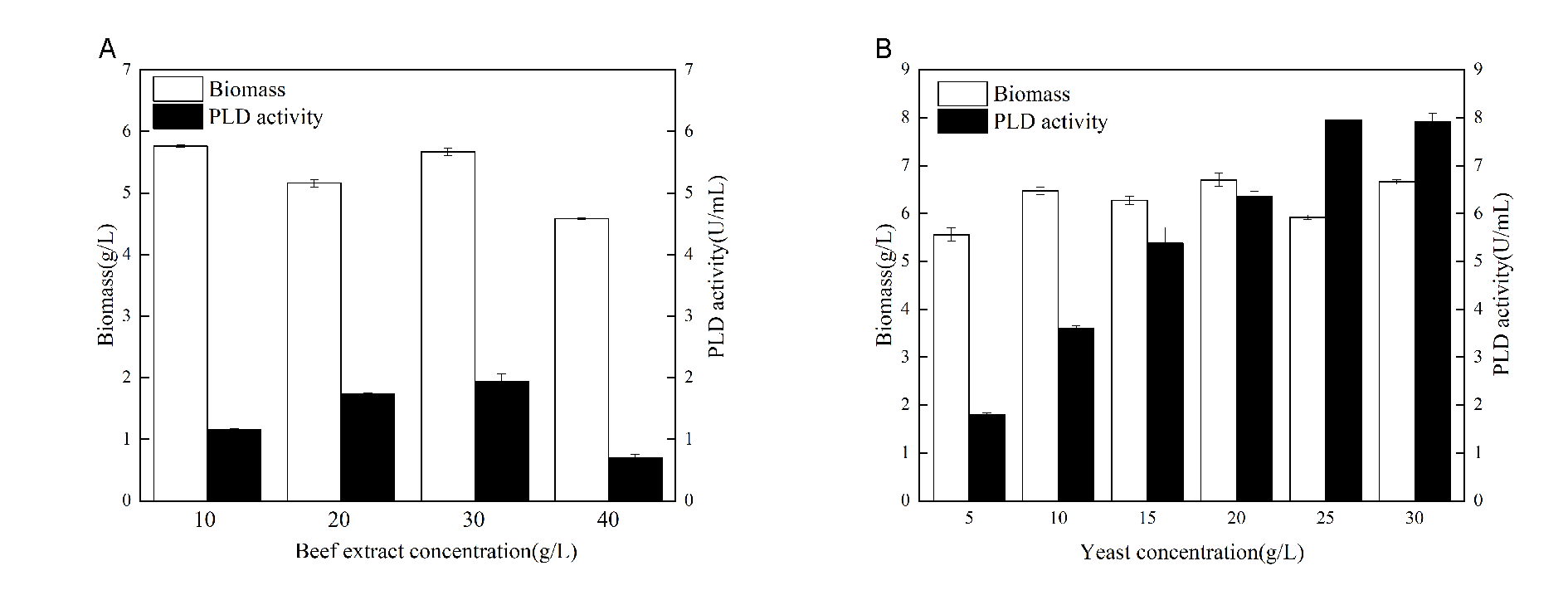


**Fig. S2** Comparison of cell growth and phospholipase D production using A. different beef extract concentrations; B. different yeast extract concentrations; Biomass (open bar), PLD activity (filled bar).

For optimizing the concentration of two nitrogen sources in fermentation medium, four different concentrations of beef extract were first tested in fermentation medium containing 5 g/L yeast extract (Fig. S2 A), and the result showed the optimum concentration of beef extract was 30 g/L. Then, five different yeast extract concentrations were further tested in fermentation medium containing the optimum beef extract concentration of 30 g/L (Fig. S2 B).


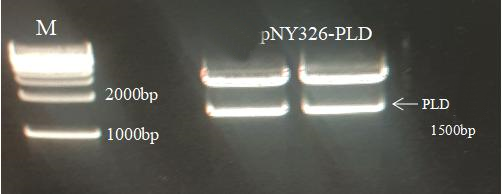


**Fig. S3** Identification of reconstructed plasmid pNY326-PLD. Lane M: DNA marker; lanes pNY326-PLD: Sample of recombinant plasmid after Xba I and EcoR I double enzyme digestion.


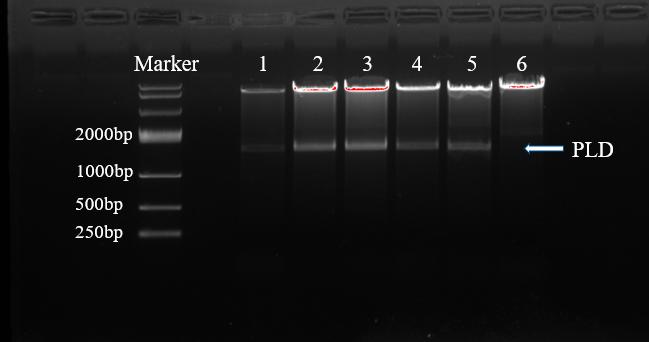


**Fig. S4** Identification of reconstructed plasmid pNCMO2-PLD. Lane Marker: DNA marker; lanes 1-6: Sample of recombinant plasmid after Xba I and EcoR I double enzyme digestion.


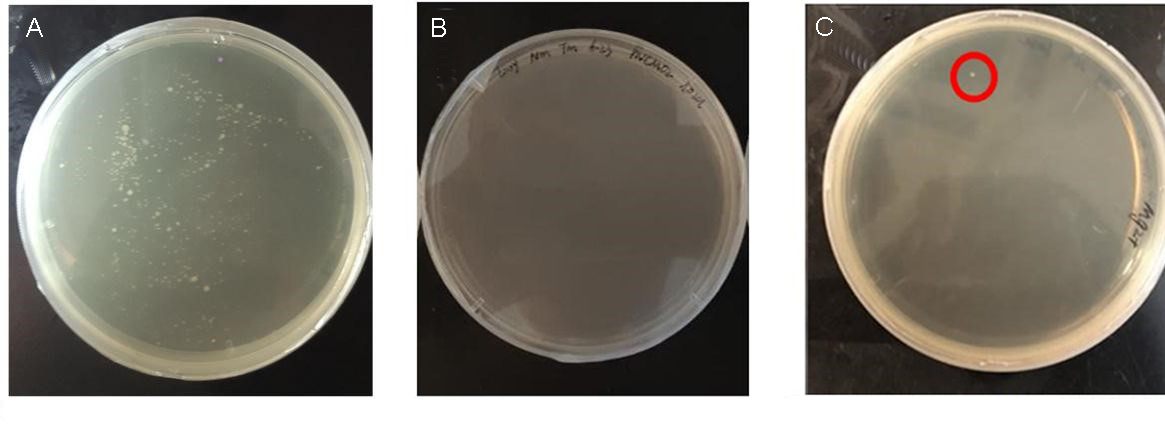


**Fig. S5** Transformation results of B. choshinensis under different conditions. Transformation of pNY326-PLD in TM with neomycin(A); transformation of pNCMO2-PLD in TM with neomycin(B); transformation of pNCMO2-PLD in TM with neomycin and Mg^2+^.


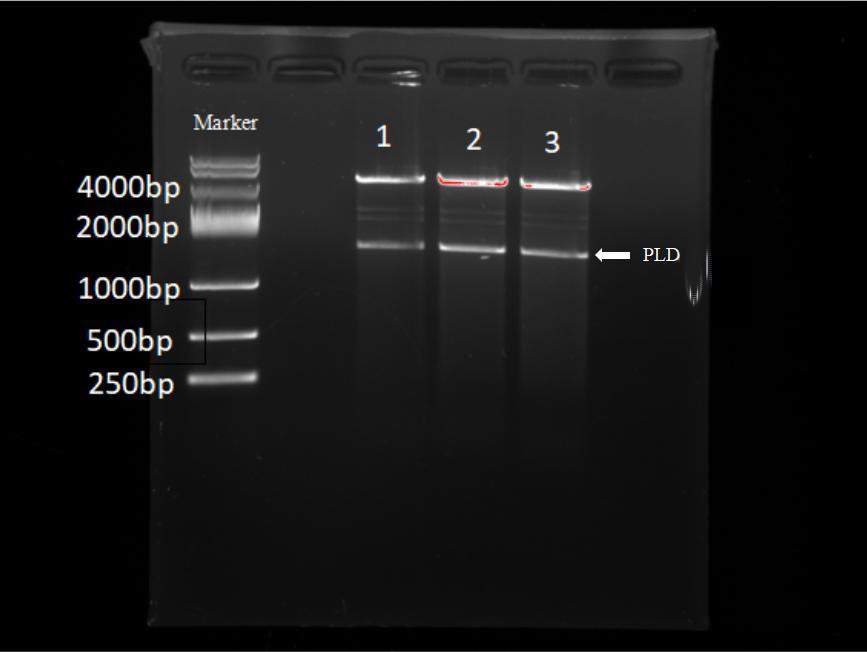


**Fig. S6** Identification of B. choshinensis/pNCMO2-PLD. Lane Marker: DNA marker; lanes 1-3: Sample of recombinant plasmid from the recombinant strains after Xba I and EcoR I double enzyme digestion.
